# Mild Fetal Tricuspid Regurgitation in the First Trimester as a Predictor of Perinatal Outcomes

**DOI:** 10.3390/medicina57060637

**Published:** 2021-06-19

**Authors:** Ji-Eun Park, Hyen-Chul Jo, Seon-Mi Lee, Jong-Chul Baek, In-Ae Cho, Soon-Ae Lee

**Affiliations:** 1 Department of Obstetrics and Gynecology, Gyeongsang National University Changwon Hospital, Changwon-si 51472, Korea; parkjieun@gnuh.co.kr (J.-E.P.); 73hccho@gnuh.co.kr (H.-C.J.); tjsal4142@gnuh.co.kr (S.-M.L.); gmfather@gnu.ac.kr (J.-C.B.); 2 College of Medicine, Gyeongsang National University, Jinju-si 52828, Korea; 3 Department of Obstetrics and Gynecology, Gyeongsang National University Hospital, Jinju-si 52727, Korea; iacho@gnuh.co.kr

**Keywords:** tricuspid regurgitation, first trimester pregnancy, prenatal ultrasonography, placenta, amniotic fluid index, fetal growth

## Abstract

*Background and Objectives*: This study aimed to investigate whether mild fetal tricuspid regurgitation (TR) at 11^+ 0^ to 13^+ 6^ weeks of gestation affects perinatal outcomes. Since fetal right ventricular load is associated with placental resistance, we hypothesized that fetal mild TR would be associated with perinatal outcomes as a consequence of abnormal placentation. *Materials and Methods*: We retrospectively evaluated 435 women with first-trimester scan data. Blood flow across the tricuspid valve was examined in singleton pregnancies between 11^+ 0^ and 13^+ 6^ weeks of gestation. Women were categorized according to the presence or absence of fetal mild TR, and the maternal and pregnancy characteristics and perinatal outcomes were compared. Multiple linear and logistic regression analyses were conducted to identify independent predictors of perinatal outcome. *Results*: In the group with mild TR, there were more cases of borderline amniotic fluid index, including oligohydramnios (*p* = 0.031), and gestational age- and sex-specific birth weights were lower (*p* = 0.012). There were no significant differences in other perinatal outcomes, including preeclampsia, gestational hypertension and small for gestational age. Gestational diabetes (adjusted odds ratio (OR) 0.514, 95% confidence interval (CI) 0.312–0.947) and fetal mild TR (adjusted OR 1.602, 95% CI 1.080–2.384) were identified as factors associated with below borderline amniotic fluid index before birth. The factors that affected gestational age and sex-specific birth weight were also gestational diabetes (adjusted beta coefficient 9.673, *p* = 0.008) and the presence of fetal mild TR (adjusted beta coefficient −6.593, *p* = 0.007). *Conclusions*: Mild fetal TR observed in the first trimester is negatively associated with fetal growth and the amniotic fluid index at term but not with other adverse pregnancy or perinatal outcomes due to abnormal placentation.

## 1. Introduction

An assessment of tricuspid flow and evaluation of tricuspid regurgitation (TR) in the first trimester of pregnancy can improve the performance of screening for aneuploidies and major cardiac defects [1,2]. In addition to the role of TR as an ultrasound marker at an early gestational age when a structural cardiac anomaly is present, one study has shown that TR is frequently used as a marker of chromosomal abnormalities in the absence of structural cardiac abnormalities, especially those that occur in trisomy 21 [3]. With improved ultrasonography (USG), the frequency of detection of fetal TR, including mild cases, observed in the first trimester of pregnancy has exceeded the previously estimated prevalence [4,5,6].

Studies have reported that the observed high prevalence of mild TR in the early stage of pregnancy suggests that it may reflect normal physiological findings [6]. However, it has been found that the fetal right ventricular afterload reflects the circulation and resistance of the placenta [7,8], and TR is observed when the right ventricular afterload increases. Moreover, TR would be a marker only in the first trimester because of the low compliance of the fetal heart and the high cardiac afterload caused by placental resistance. Therefore, in the presence of uteroplacental insufficiency, the right ventricular afterload increases due to increased placental resistance, which can lead to TR.

We hypothesized that mild TR, which appears in the first trimester of pregnancy, in the absence of fetal chromosomal and heart abnormalities, may play a role as an early marker of increased placental resistance due to placental problems. To evaluate this hypothesis, we evaluated pregnancy and perinatal outcomes, including fetal birth weight, according to the presence or absence of mild TR. The aim of this study was to investigate the association between mild TR and pregnancy and perinatal outcomes.

## 2. Materials and Methods

### 2.1. Study Design and Participants

**Figure 1:**
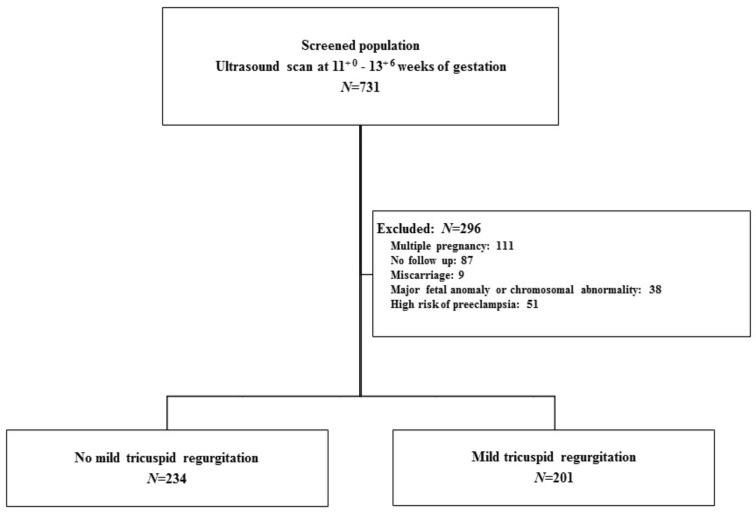
Description of the study population.

Retrospective evaluation of our records revealed that 731 women underwent TR assessment during first-trimester ultrasonography between October 2016 and December 2019. The inclusion criteria were women with singleton pregnancies who were undergoing ultrasound examinations involving measurement of blood flow across the fetal tricuspid valve at 11^+ 0^ to 13^+ 6^ weeks of gestation and whose follow-up and deliveries were performed in the study institution and who had accessible medical records. The exclusion criteria were multiple pregnancies, follow-up loss, abortion before 20 weeks of gestation, fetal malformation or chromosomal abnormality and high risk of preeclampsia (history of preeclampsia, chronic hypertension, pregestational diabetes mellitus, renal disease or autoimmune disease). According to our criteria, a total of 435 eligible pregnant women were invited to be enrolled in the study (Figure 1). 

### 2.2. Procedure

**Figure 2:**
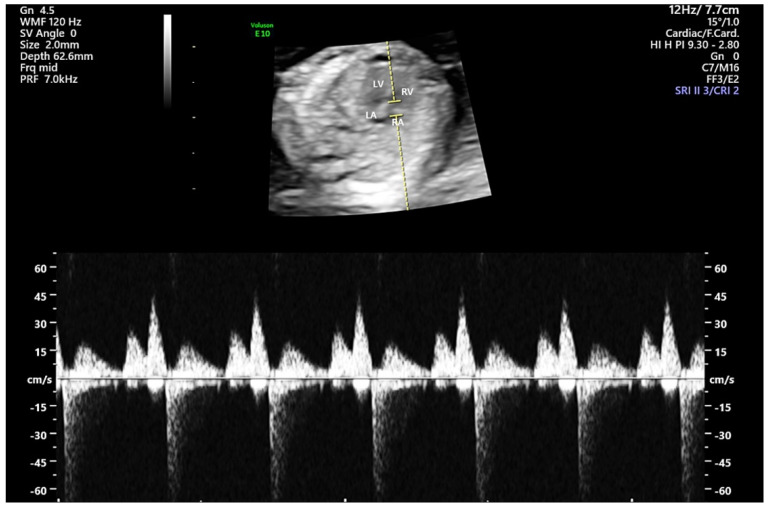
Pulse Doppler trace of fetal mild tricuspid regurgitation. LV, left ventricle; RV, right ventricle; LA, left atrium; RA, right atrium.

All eligible women underwent routine ultrasonography scans at 11^+ 0^ to 13^+ 6^ weeks of gestation using a Samsung Elite WS80A (Samsung Medison Co, Ltd., Seoul, Korea) or GE Voluson E10 (GE Healthcare Austria GmbH & Co, OG, Zipf, Austria) machine. Most examinations were performed transabdominally; nevertheless, in selected cases, transvaginal ultrasound was employed to complete the examination. Ultrasound examination was performed to assess the fetal crown–rump length, fetal nuchal translucency thickness and blood flow across the tricuspid valve. For the diagnosis of TR to be made, the magnification of the image should be such that the fetal thorax occupies most of the image, an apical four-chamber view of the fetal heart is obtained, the angle to the direction of flow is less than 30° from the direction of the interventricular septum [1] and a pulsed-wave Doppler sample volume of 2.0–3.0 mm is positioned across the tricuspid valve or the size of the color box is set to 60 cm/s when possible (Figure 2). Only waveforms consisting of clear E (early diastole) and A waves (atrial contraction in late diastole) during fetal quiescence were accepted. The diagnosis of mild TR was made according to a velocity of more than 30 cm/s and less than 70 cm/s [9] or when the jet length to the atrium (distance from the tricuspid valve to the opposite atrial wall) was less than 1/3 [6]. The evaluation of the tricuspid valve was performed by two experienced obstetricians with more than 10 years of prenatal diagnostic ultrasound experience who performed fetal echocardiography. During all Doppler examinations, the as low as reasonably achievable (ALARA) principle was followed [10].

Data regarding maternal characteristics were gathered from electronic medical records. We extracted the following data: maternal age at delivery, maternal height, maternal prepregnancy weight, gestational weight gain, maternal weight at birth, maternal body mass index (BMI) at birth, nuchal translucency, flow velocity measurement through the tricuspid valve and value of maternal serum PAPP-A level at 11^+ 0^ to 13^+ 6^ weeks of gestation.

All women received periodic prenatal assessments and delivered at the same hospital. Pregnancy and perinatal outcome data were collected from relevant electronic databases. Pregnancy and perinatal outcomes included preeclampsia, gestational hypertension, gestational diabetes, stillbirth, amniotic fluid index in the last prenatal ultrasound exam, gestational age at birth, birth weight, sex, Apgar scores and placental weight. Borderline amniotic fluid index (AFI) was defined as 5.1–8 cm, and oligohydramnios was defined as <5 cm. Gestational age- and sex-specific birth weight percentiles were transformed for each neonate based on derived Korean growth curves, which had been validated against normal intrauterine growth patterns. Small for gestational age (SGA) was defined as a birth weight of less than the 10th percentile for gestational age. According to the American College of Obstetricians and Gynecologists (ACOG) diagnostic criteria [11], gestational hypertension was defined as a blood pressure ≥140/90 mmHg without proteinuria or with proteinuria of no greater than trace levels after 20 weeks of gestation. Preeclampsia was defined as a blood pressure ≥140/90 mmHg with proteinuria of 1+ on dipstick in two samples taken 6 h apart or >0.3 g in a 24-h urine collection.

### 2.3. Statistical Analysis

Statistical analysis was performed using ANOVA and *t*-tests when comparisons were made between the groups and when the data were normally distributed. If the data were not normally distributed, the nonparametric Kruskal–Wallis test and Mann–Whitney U test were chosen. Categorical data were compared using the chi-square test or Fisher’s exact test. The crude odds ratio and 95% confidence interval were calculated for all factors studied in the analysis. Multiple linear and logistic regression analyses were conducted to determine independent predictors of pregnancy outcome. Factors identified as associated in the univariate analysis at a level of less than 0.1 were included in this stepwise procedure. Statistical significance was defined as a *p*-value <0.05. Statistical analyses were performed using R 4.0.3.

### 2.4. Ethics

The study protocol and the waiver of informed consent were approved by the Institutional Review Board (IRB) of Gyeongsang National University Changwon Hospital (serial number: GNUCH 2019-07-029). All methods were performed in accordance with the relevant guidelines and regulations of the institution.

## 3. Results

A total of 435 pregnant women were enrolled in the study. The mean maternal age at pregnancy was 33.0 (±4.5) years, the mean gestational age was 37.9 (±1.9) weeks, the mean AFI at the last prenatal ultrasound exam was 9.5 (±3.7) and the mean gestational age- and sex-specific birth weight percentile was 52.5 (±26). A total of 201 (46%) cases of mild TR in the first trimester of pregnancy were observed. The maternal and obstetric characteristics and perinatal outcomes of those with and without fetal mild TR in the first trimester of pregnancy are described in Table 1. In the mild TR group, tricuspid E and A wave velocities were high, and there was no difference in the tricuspid E/A ratio between the two groups. The number of cases of below borderline AFI, including oligohydramnios, was larger in the mild TR group (*p* = 0.031). The gestational age- and sex-specific birth weight percentiles were lower in the mild TR group (*p* = 0.012). Other maternal and obstetric characteristics and the frequencies of adverse pregnancy outcomes, namely, preeclampsia, gestational hypertension, gestational diabetes and stillbirth, were not different between the groups.

We performed logistic regression to identify factors associated with less than borderline AFI before birth. The variables that exhibited significant differences in univariate analysis were further evaluated with the backward stepwise logistic regression method. The analysis revealed that the odds ratios of the presence of gestational diabetes mellitus (GDM) and mild TR for less than borderline AFI before birth were 0.514 (0.312–0.947 95% CI, *p* = 0.038) and 1.602 (1.080–2.384 95% CI, *p* = 0.019), respectively (Table 2).

We performed univariate and multivariate linear regression analyses to identify independent factors that affected fetal growth in terms of gestational age- and sex-specific birth weight. The presence of GDM (β = 9.673, 2.554–16.792 95% CI, *p* = 0.008) and mild TR (β = 6.593, −11.38–−1.805 95% CI, *p* = 0.007) remained independently associated with fetal growth. No significant correlation was observed between maternal age, weight and weight change during pregnancy and gestational age- and sex-specific birth weight (Table 3).

Whether the presence of mild TR was associated with other unfavorable perinatal outcomes was analyzed using multiple linear and logistic regression analyses, but no statistically significant association was found.

## 4. Discussion

The findings of this study demonstrate that mild TR, which was observed in the first trimester of pregnancy, was negatively associated with amniotic fluid and fetal growth. However, the results showed that mild TR was not correlated with adverse pregnancy or perinatal outcomes associated with the placenta, contrary to our hypothesis.

Studies on the prevalence of mild TR in early fetal life are lacking. In this study, mild TR in the first trimester of pregnancy was found in 46% (201/435) of cases. Earlier studies have reported that the prevalence of TR in low-risk fetal populations (without chromosomal abnormalities and structural abnormalities) is 1.7–6.23%, although the class or degree of TR was not clearly defined in these studies [4,5]. We speculate that the main reason for this discrepancy is that previous TR diagnostic criteria were limited when the regurgitation jet velocity was at least 60 cm/s and its interval was extended to more than half of systole. In addition, we speculate that the difference in the period during which TR was confirmed will also have an effect. We studied only TR observed between 11 and 13 weeks of gestation, but previous studies have targeted the entire gestational period or the second trimester. On the other hand, a recent study using the spatiotemporal image correlation (STIC) technique showed a high prevalence (83.4%) of mild TR from 11–14 weeks of gestation, with a significant decrease (24.8%) at the time of the mid-trimester scan [6]. This difference is presumed to be due to the high sensitivity of the STIC technique.

There is a lack of research on the relationship between TR that is not associated with chromosomal or structural abnormalities and pregnancy and perinatal outcomes. However, according to previous reports, fetal circulation occurs in parallel, and the fetal right ventricle pumps against the systemic pressures of the lower fetal body and placental impedance, while the left ventricle ejects against the relatively high impedance of the fetal brain and upper body [7,8,12]. The earlier in pregnancy, the greater the portion of placental resistance that constitutes the afterload. The afterload is dominated by placental resistance, which is high in early pregnancy and decreases after the first trimester [3]. Mild TR of the right heart can be used as a sensitive index of placental resistance. Mäkikallio et al. [7] reported that the incidence of TR was increased when there was retrograde blood flow in the fetal aortic isthmus with placental insufficiency and/or fetal growth restriction. On the contrary, Gembruch and Smrcek [5] investigated the relationship between TR and intrauterine growth restriction and reported no significant difference in the prevalence of TR between fetuses with intrauterine growth restriction and those with normal growth. In our study, the high SGA incidence in the group with mild TR was not statistically significant, but gestational age- and sex-specific birth weight was high in the group without TR and lower in the group with TR. In addition, even if maternal age and weight, maternal BMI, the amount of weight gained during pregnancy and gestational diabetes are adjusted, which have been found to have a great influence on the size of the fetus [13,14], the effect of TR on gestational age- and sex-specific birth weight is meaningful.

Research results are divergent as to whether borderline AFI is associated with poor perinatal outcomes [15,16]. Although the factors affecting amniotic fluid volume are complex, it is well known that decreased amniotic fluid reflects chronic placental insufficiency, which occurs due to fetal oliguria due to blood flow redistribution [17]. In our study, the TR group showed a high percentage of borderline low amniotic fluid before birth. Our study showed that in the group with mild TR, there was a higher incidence of less than borderline AFI before birth than in the group without mild TR.

Poor placentation means that trophoblast invasion is inhibited, the spiral arteries are poorly remodeled and the capacity of uteroplacental circulation is too limited. After 9 weeks of gestation, the uteroplacental arteries recanalize from the placental periphery and are completed by 12 weeks. Afterward, invasive cytotrophoblasts in decidual tissue extensively remodel the spiral arteries [18]. This poor placentation is known to be associated with preeclampsia or SGA [19]. Thus, we predicted that the presence of mild TR observed in the first trimester would be associated with poor placentation and that it would be associated with preeclampsia; however, the results of this study showed that there was no significant difference in the prevalence of preeclampsia or placental weight according to the presence of TR.

The main limitation of our study is its retrospective, single-center design and small sample size. Large-scale multicenter studies are needed to obtain meaningful obstetric and perinatal outcome data that depend exclusively on the presence or absence of mild TR without other risk factors. Another limitation is that even if two obstetricians consecutively identify mild TR, the confirmation of fetal TR is heavily dependent on the proficiency of the observer, the machine settings and the technique used (ex-STIC vs. pulse wave vs. color mapping). This will always be a limitation of research in the context of prenatal ultrasonography. In addition, we did not observe changes in mild TR, that is, during follow-up as gestation progressed. If these changes over time and other factors related to pregnancy and perinatal outcomes (in other words, parameters related to placental pathophysiology or observational variables, such as poor obstetrical history) were included in the study design, the results of this study would have been more complete. Therefore, it would be worthwhile to perform prospective, well-designed follow-up studies in the future.

However, this study is meaningful as an early study to address the meaning of mild fetal TR in the first trimester and its role in predicting pregnancy and perinatal outcomes. Based on an understanding of the pathophysiology of the placenta and the cardiovascular system of the fetus, we expect more clinically meaningful results based on follow-up research in the future.

## 5. Conclusions

Fetal mild TR observed at 11–13 weeks of gestation is associated with fetal growth and AFI at term but not with adverse pregnancy or perinatal outcomes due to abnormal placentation. Nevertheless, developing a predictive tool to use in early pregnancy to predict future fetal growth abnormalities, amniotic fluid abnormalities occurring during late pregnancy or unfavorable perinatal outcomes is very innovative and important. Moreover, screening for mild TR in early pregnancy can select cases that require more careful prenatal care in late pregnancy, when problems with fetal weight gain or amniotic fluid balance may occur, which suggests that this screening can be beneficial in improving pregnancy outcomes and counseling. Therefore, this study is very meaningful as an early study in this direction.

## Figures and Tables

**Table 1:** Comparison of maternal and obstetric characteristics and perinatal outcomes between groups according to the presence of mild tricuspid regurgitation in the first trimester of pregnancy.

	No Mild TR (*n* = 234)	Mild TR (*n* = 201)	*p*-Value
Age (years)	32.8 ± 4.5	33.3 ± 4.5	0.198
Maternal weight at birth (kg)	70.6 ± 10.4	70.2 ± 11.4	0.676
Maternal BMI at birth (kg/m^2^)	22.5 ± 5.2	22.5 ± 3.5	0.899
Maternal prepregnancy weight (kg)	58.4 ± 11.1	58.8 ± 10.6	0.713
Gestational weight gain	12.2 ± 5.7	11.0 ± 7.4	0.067
TR velocity (cm/s)	**11.5 ± 5.3**	**30.1 ± 10.8**	**<0.001**
Tricuspid E wave velocity (cm/s)	**24.1 ± 6.0**	**29.1 ± 8.3**	**<0.001**
Tricuspid A wave velocity (cm/s)	**45.5 ± 8.8**	**53.2 ± 12.8**	**<0.001**
Tricuspid E/A	**0.5 ± 0.1**	**0.5 ± 0.1**	**0.029**
Nuchal translucency (mm)	1.7 ± 0.8	1.6 ± 0.8	0.867
PAPP-A (MoM)	1.2 (0.6; 1.8)	1.2 (0.6; 1.8)	0.43
Gestational diabetes	24 (42.1%)	33 (57.9%)	0.079
AFI before birth	9.5 ± 3.4	9.6 ± 4.0	0.829
Oligohydramnios (AFI < 5 cm)	20 (43.5%)	26 (56.5%)	0.184
< Borderline AFI (AFI < 8 cm)	**80 (47.1%)**	**90 (52.9%)**	**0.031**
Preeclampsia	8 (50.0%)	8 (50.0%)	0.956
Gestational hypertension	5 (55.6%)	4 (44.4%)	1
Stillbirth	1 (50.0%)	1 (50.0%)	1
Sex of the neonate			0.721
Male	125 (54.8%)	103 (45.2%)	
Female	109 (52.7%)	98 (47.3%)	
Gestational age at birth (weeks)	**38.1 ± 1.8**	**37.7 ± 2.0**	**0.022**
Birth weight (g)	**3124.5 ± 453.3**	**2978.2 ± 528.1**	**0.001**
Birth weight percentile	**55.4 ± 24.8**	**49.1 ± 26.9**	**0.012**
Small for gestational age	6 (40.0%)	9 (60.0%)	0.408
Apgar score at 1 min	8.0 ± 1.0	8.0 ± 1.1	0.434
Apgar score at 5 min	8.62 ± 0.75	8.57 ± 0.85	0.511
Placental weight (g)	713.5 ± 137.2	697.7 ± 139.0	0.135

TR: tricuspid regurgitation; BMI: body mass index; PAPP-A: pregnancy-associated plasma protein-A; AFI: amniotic fluid index. Data are shown as the mean ± SD or *n* (%). The bold values are values with significant differences (*p* < 0.05).

**Table 2:** Association between factors and borderline AFI according to logistic regression analysis.

	Factors Associated with Borderline AFI			
	Crude		Adjusted	
	OR (95% CI)	*p*-Value	OR (95% CI)	*p*-Value
Age	1.033 (0.989–1.079)	0.142	1.033 (0.988–1.080)	0.155
Maternal weight at birth	1.008 (0.990–1.026)	0.392	1.231 (0.264–NA)	0.978
Maternal prepregnancy weight	1.005 (0.987 1.022)	0.605	0.827 (NA–3.777)	0.98
Maternal BMI at birth	1.001 (0.957–1.045)	0.979	0.968 (0.852–1.046)	0.505
Gestational weight gain	0.999 (0.970–1.030)	0.934	0.825 (NA–3.883)	0.98
Gestational diabetes	0.568 (0.300–1.029)	0.07	**0.514 (0.312–0.947)**	**0.038**
Mild TR	1.561 (1.060–2.303)	0.024	**1.602 (1.080–2.384)**	**0.019**

NA: not available; AFI: amniotic fluid index; BMI: body mass index; TR: tricuspid regurgitation; OR: odds ratio; CI: confidence interval. The bold values are values with significant differences (*p* < 0.05).

**Table 3:** Association between factors and birthweight percentile by linear regression analysis.

	Factors Associated with Birthweight Percentile			
	Crude		Adjusted	
	Beta Coefficient (95% CI)	*p*-Value	Beta Coefficient (95% CI)	*p*-Value
Age	0.163 (−0.384, 0.71)	0.557	0.071 (−0.465, 0.607)	0.795
Maternal weight at birth	0.532 (0.312, 0.753)	0	0.194 (−0.631, 1.02)	0.644
Maternal prepregnancy weight	0.467 (0.246, 0.688)	0	0.371 (−0.503, 1.244)	0.404
Maternal BMI at birth	0.802 (0.259, 1.346)	0.004	−0.186 (−1.046, 0.673)	0.67
Gestational weight gain	0.28 (−0.095, 0.655)	0.143	0.325 (−0.378, 1.028)	0.364
Gestational diabetes	9.764 (2.561, 16.966)	0.008	**9.673 (2.554, 16.792)**	**0.008**
Mild TR	−6.291 (−11.17, −1.412)	0.012	**−6.593 (−11.38, −1.805)**	**0.007**

TR: tricuspid regurgitation; OR: odds ratio; CI: confidence interval. The bold values are values with significant differences (*p* < 0.05).

## Data Availability

No new data were created or analyzed in this study. Data sharing is not applicable to this article.
